# Neural side effect discovery from user credibility and experience-assessed online health discussions

**DOI:** 10.1186/s13326-020-00221-1

**Published:** 2020-07-08

**Authors:** Van-Hoang Nguyen, Kazunari Sugiyama, Min-Yen Kan, Kishaloy Halder

**Affiliations:** School of Computing, National University of Singapore, 13 Computing Drive, Singapore, 117417 Singapore

**Keywords:** Online health communities, Drug side effect discovery, Credibility analysis, Deep learning, Natural language processing

## Abstract

**Background:**

Health 2.0 allows patients and caregivers to conveniently seek medical information and advice via e-portals and online discussion forums, especially regarding potential drug side effects. Although online health communities are helpful platforms for obtaining non-professional opinions, they pose risks in communicating unreliable and insufficient information in terms of quality and quantity. Existing methods in extracting user-reported adverse drug reactions (ADRs) in online health forums are not only insufficiently accurate as they disregard user credibility and drug experience, but are also expensive as they rely on supervised ground truth annotation of individual statement. We propose a NEural ArchiTecture for Drug side effect prediction (NEAT), which is optimized on the task of drug side effect discovery based on a complete discussion while being attentive to user credibility and experience, thus, addressing the mentioned shortcomings. We train our neural model in a self-supervised fashion using ground truth drug side effects from mayoclinic.org. NEAT learns to assign each user a score that is descriptive of their credibility and highlights the critical textual segments of their post.

**Results:**

Experiments show that NEAT improves drug side effect discovery from online health discussion by 3.04*%* from user-credibility agnostic baselines, and by 9.94*%* from non-neural baselines in term of *F*_1_. Additionally, the latent credibility scores learned by the model correlate well with trustworthiness signals, such as the number of “thanks” received by other forum members, and improve credibility heuristics such as number of posts by 0.113 in term of Spearman’s rank correlation coefficient. Experience-based self-supervised attention highlights critical phrases such as mentioned side effects, and enhances fully supervised ADR extraction models based on sequence labelling by 5.502*%* in terms of precision.

**Conclusions:**

NEAT considers both user credibility and experience in online health forums, making feasible a self-supervised approach to side effect prediction for mentioned drugs. The derived user credibility and attention mechanism are transferable and improve downstream ADR extraction models. Our approach enhances automatic drug side effect discovery and fosters research in several domains including pharmacovigilance and clinical studies.

## Background

Seeking medical opinions from online health communities has become popular: 71% of adults aged 18–29 (equivalent to 59% of all U.S. adults) reported consulting online health websites for opinions [1]. These opinions come from an estimated twenty to one hundred thousand health-related websites [2], inclusive of online health communities that network patients with each other to provide information and social support [3]. Platforms such as HealthBoards^1^ and MedHelp^2^ feature users reporting their own health experiences, inclusive of their self-reviewed drugs and medical treatments. Hence, they are valuable sources for researchers [4, 5].

Although patients use these platforms to access valuable information about drug reactions, there are challenges to their effective, large-scale use. There is lexical variation where users describe the same side effect differently. For example, *dizziness* can be expressed as *giddiness* or *my head is spinning*, posing difficulty to most feature-based or keyword matching approaches. Separately, there are valid concerns regarding credibility of user-generated contents to be harvested at large in which research has shown to be of variable quality and should be approached with caution [6–9]. One proxy indicator for information quality is the author’s trustworthiness [10]. In the context of social media or online forums, user trustworthiness is often approximated via ratings from other users, i.e., number of thanks or upvotes [11], or via their consistency of reporting credible information [12, 13]. In addition to credibility, forum members also offer expertise thanks to their own experience – with prescriptions in particular – and facilitate responses to drug queries [14]. For instance, while reporting expected side effects for a specific treatment, patients with long-term use of certain drugs can be a complementary source of information:

*While my experience of 10 years is with Paxil, I expect that Zoloft will be the same. You should definitely feel better within 2 weeks. One way I found to make it easier to sleep was to get lots of exercize [sic]. Walk or run or whatever to burn off that anxiety.* – User 3690.

The above is an answer to a thread asking for expected side effects for depression treatment with Zoloft. User 3690’s history of active discussion on other anti-depressants such as Lexapro and Xanax lends credibility to them being an authority on depression treatments. We noticed that Zoloft (mentioned in the thread) shares many common side effects with the other two anti-depressants: “*changed behavior*,” “*dry mouth*,” and “*sleepiness or unusual drowsiness*.” as illustrated in Table 1. Many such examples suggest that drugs which are often prescribed together for the same treatment, such as anti-depressants, are likely to be discussed within a same thread and share common side effects. In addition, users who have experienced certain drug reactions are more outspoken and active on those discussions involving drugs of similar side effects. These signals arise from the rich context of online health information; hence, we expect systems to explore beyond individual statements. Specifically, they should consider the complete discussion content as well as the global experience of each involved users, in order to discover drug side effects or extract adverse drug reactions (ADRs).

**Table 1:** Side effects of anti-depressants

Drugs	Side effects
Lexapro	chills, constipation, cough, decreased appetite, **decreased sexual desire**, **diarrhea**, **dry mouth**, joint pain, muscle ache, tingling feeling, **sleepiness or unusual drowsiness**, unusual dream, **sweating**,...
Xanax	abdominal or stomach pain, muscle weakness, **changed behavior**, chills, cough, decreased appetite, decreased urine, **diarrhea**, difficult bowel movement, cough, **dry mouth**, tingling feeling, **sleepiness or unusual drowsiness**, slurred speech, sweating, yellow eye,..
Zoloft	**changed behavior**, decreased sexual desire, **diarrhea**, **dry mouth**, heartburn, **sleepiness or unusual drowsiness**, **sweating**,..

The Drugs and Side effects columns respectively list the anti-depressants and their side effects extracted from a drug–side effect database. Side effects in common among those listed are bold

We argue that modeling user expertise from experienced side effects is more robust compared against general user profile and engagement features [13, 14], as user expertise provides more meaningful signals for side effect discovery. To the best of our knowledge, there is no previous work that incorporates user expertise in side effect discovery in discussion forums at either the thread or post level. In this work, given online health discussions, we propose a novel end-to-end neural architecture that jointly models each author’s credibility, their global experience and their post’s textual content to discover the side effect of unseen drugs. We optimize the model on a self-supervised task of predicting side effect of mentioned drugs for complete threads, where ground truth is accessible. Our key observation is that users can be grouped into clusters that share the same expertise or interest in certain drugs, possibly due to their common treatment or medical history. We incorporate this critical observation into our user model in representing a post’s content via a cluster-sensitive attention mechanism [15]. We also follow general definition of truth discovery and let the model learn a credibility score that is unique to every user and descriptive of their trustworthiness. Our experiments include an overall ablation study to validate the significance of each model component. This paper extends our former work [16] by conducting a correlation study that analyzes the representativeness of learned credibility scores and a comparison between our self-supervised attention-based approach and traditional supervised sequence labeling approaches on side effect mention extraction.

We summarize our contributions as follows:

- We propose a NEural ArchiTecture, NEAT, that captures 1) user expertise and 2) credibility, 3) the semantic content of individual posts and 4) the complete discussion thread, to improve side effect discovery from online health discussions. NEAT’s main means of user credibility and experience assessment can be easily adopted by various neural attentional encoders [17, 18].
- We formulate a self-supervised task of side effect prediction of mentioned drugs for the proposed network to jointly optimize its components.
- We conduct experiments to verify the validity of our learned credibility and the robustness of self-supervised attention-based extraction, comparing against traditional supervised sequence labeling baselines.

## Related work

We first review existing approaches to drug side effect discovery from health forums and social media. Next, we examine how these works incorporate user credibility and expertise in their learning objective. Finally, we justify our choice of neural architecture by discussing its modeling capability of context-rich structures such as online discussion.

**Drug Side Effect Discovery.** Existing methods for drug discovery from online content extract drugs at post and statement level. ADR mining systems typically include a named entity recognition (NER) model and a relationship or semantic role labeling model [19, 20]. Recent neural approaches address lexical variation in user-generated content – the difficulty faced by traditional keyword matching and rule-based approaches – to improve recognition and labeling components [21, 22]. Distributed word representations [23, 24] constructed from context can capture semantics based on the hypothesis that synonyms often share similar contextual words. For example, “headache” and “cephalea” will have close representations if they share contextual words such as “head” or “pain”. Approaches to sub-word embedding [25, 26] model the morphology of words by leveraging sub-word or character information. These representations are naturally integrated into neural sequential models [17, 18, 27] that are sensitive to syntactic order. However, supervised sequence labeling or mention extraction approaches require laborious annotations at the word (token) level, and are only capable of discovering side effects that are explicitly present in the text. Expert supervision or additional semantic matching models are also required to map such recognized text segments to standardized vocabularies or thesaurii [28]. In contrast, our proposed self-supervised task formulation discovers the aggregated side effects of mentioned drugs for each community discussion by considering the whole thread’s content. The list of discussed drugs are tagged by forum moderators or obtained by pattern matching. This learning design not only effectively alleviates the need for expensive, finer-grained annotations but also allows for the prediction of side effects not explicitly mentioned in the discussion.

**User Credibility and Expertise Integration.** Credibility is of the utmost concern in large-scale knowledge harvesting [8, 29, 30]. Previous work on side effect discovery from individual statements or posts derive information credibility by verifying a statement’s mentioned side effects against ground truth drug side effect databases, and assess associated user credibility by measuring the percentage of a user’s credible statements [13, 31]. In contrast, our approach to side effect discovery from discussions by jointly modeling multiple posts and authors eschews the assessment of statement credibility and derives user credibility differently. We assign each user a positive score that is used to weight their post content in representing the discussion’s holistic content. Such weighted summation is detailed mathematically in Appendix 1 to conform to the general principle of truth discovery, where sources providing credible information should be assigned higher credibility scores, and the information that is supported by credible sources will be regarded as true [10]. Although our dataset does not provide any ground truth for user trustworthiness, we followed the previous usage of ratings or upvotes in online forums and adopted the number of “thanks” received from other forum members [11] as our proxy for user trustworthiness. Previous works have modeled user expertise based on user profiles such as demographics; activity features such as posting frequency and posting pattern through time series and network analysis [13, 14]. As shown in an earlier example in Section 1, modeling user expertise from previously experienced side effects better captures author authoritativeness for certain side effects. It is also universally applicable to any online platform.

**Modeling Online Discussion Content and Structure** As our work makes use of the rich topographical properties of online communities, we briefly review approaches for modeling textual content and post-thread discussion structure. Previous works use probabilistic graphical models implicitly to represent textual content (especially, topic modeling) as bags-of-words [28, 32] or inventories of stylistic and linguistic features [13]. Such lightweight representation are well-suited in moderately short contexts, i.e., sentences or posts. However, in terms of modeling long discussions consisting of multiple posts, state-of-the-art models for Community Question Answering (CQA) feature hierarchical neural architectures [33–35]. In term of encoding text, sequential encoders such as Long Short-Term Memory (LSTM) [36] or Convolutional Neural Networks (CNN) [18] are capable of encoding long-term dependencies and semantic expressiveness by leveraging word embeddings. In terms of encoding hierarchical structures such as community discussions consisting of post- and thread-level features, neural architectures allow for straightforward and efficient integration of multiple learning objectives. In addition, our neural architecture, NEAT, incorporates attention mechanism that focuses on essential phrases while encoding post content, and joint user credibility learning while optimizing for the side effect discovery objective.

## Methods

**Basic Terminology.** To ensure a consistent representation, we define some terms and formalize them as follows:

- A *drug d* has a set of side effects,
  $S_{d} = \{s_{1}, s_{2}, \ldots,s_{|S_{d}|}\}\phantom {\dot {i}\!}$
- A *post p* is a message in online forums and contains a sequence of words. Each post *p* belongs to the set of all online forum posts *P* and is written by a *user u* and belongs to a *thread t*.
- A *user u* is a member of an online forum and participates in a list of threads, i.e., $T_{u} = \{t_{1}, t_{2}, \ldots, t_{|T_{u}|}\}\phantom {\dot {i}\!}$ by writing at least one post in each thread. We use the terms *user* and *author*, as well as *user experience* and *user expertise* interchangeably. Each user belongs to the set of all online forum users *U* and is characterized by their credibility and expertise. Credibility *w*_*u*_ of user *u* reflects the probability of user *u* provide trustworthy or helpful information, and is approximated from the number of “thanks” given from other forum members.
- A *thread t* (see Table 2) is an ordered collection of post–user pairs,

**Table 2:** A sample discussion thread from an online health community

User IDs	Posts	Mentioned drugs	Aggregated side effects
3690	While my experience of 10 years is with Paxil, I expect that Zoloft will be the same. You should definitely feel better within 2 weeks. One way I found to make it easier to sleep was to get lots of exercize. Walk or run or whatever to burn off that anxiety.	Zoloft, Paxil	changed behavior, decreased sexual desire, diarrhea, dry mouth, heart-burn, sleepiness or unusual drowsiness,...
26521	I’ve heard of people going “cold turkey” and having withdrawal at 6 months! Please, get in contact with a doctor ASAP! “common symptoms include dizziness, electric shock-like sensations, sweating, nausea, insomnia, tremor, confusion, nightmares and vertigo”		

The User IDs and Posts columns respectively list the IDs of users involved in the discussions and their messages. The Mentioned drugs and Aggregated side effects columns respectively list the explicitly discussed drugs and their combined side effects
  $Q_{t} = \{\left (p_{1}, u_{1}\right), \left (p_{2}, u_{2}\right), \ldots, \left (p_{|Q_{t}|}, u_{|Q_{t}|}\right)\}$.
  Each thread discusses the treatment for a particular condition and entails a list of prescribed drugs $\phantom {\dot {i}\!}D_{t}=\{d_{1}, d_{2}, \ldots, d_{|D_{t}|}\}$. Hence, every thread has a list of aggregated potential side effects defined as $\phantom {\dot {i}\!}S_{t} = S_{d_{1}} \cup S_{d_{2}} \cdots \cup S_{d_{|D_{t}|}}$.

**Task Definition.***Drug side effect discovery from discussions* is the task of assigning the most relevant subset of potential side effects to threads discussing certain drugs, from a large collection of side effects. We view the drug side effect discovery problem as a multi-label classification task. In our setting, an instance of item–label is a tuple (***x***_*t*_,***y***) where ***x***_*t*_ is the feature vector of thread *t* derived from its list of post–user pairs *Q*_*t*_ and ***y*** is the side effect label vector i.e., ***y***∈{0,1}^|*S*|^, where |*S*| is the number of possible side effect labels. Given training instances, we train our classifier to predict the list of drug side effects in unseen threads discussing unseen drugs.

**Formal Hypothesis.** Given a thread *t* with *Q*_*t*_, we hypothesize that considering the credibility and experience of user *u*∈(*p*,*u*)∈*Q*_*t*_ improves the quality of feature representation in thread *t*, resulting in better drug side effect discovery performance.

**Fig. 1:**
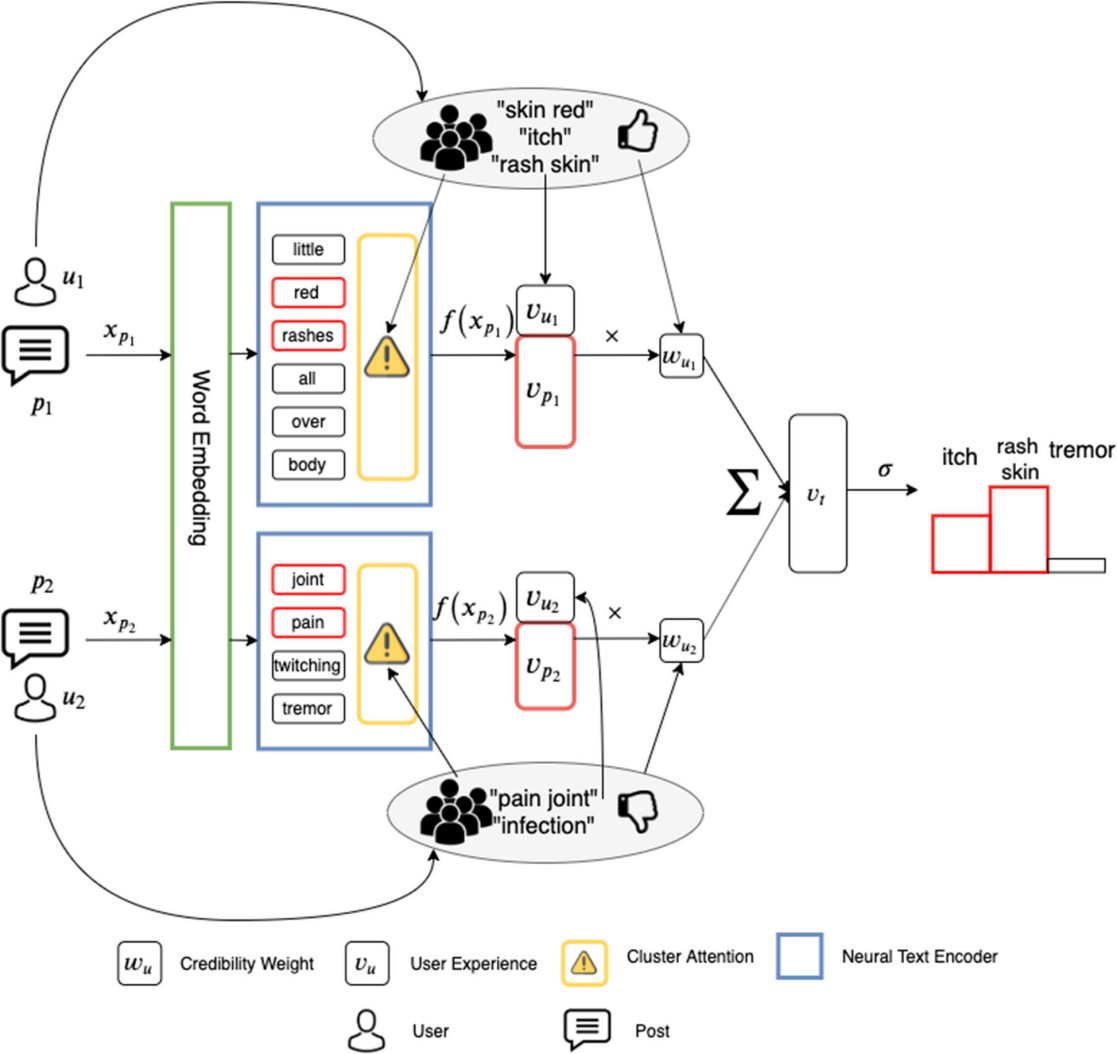
The neural architecture of our proposed NEAT. The *w*_*u*_ and *v*_*u*_ boxes denote Credibility Weight (CW) component and User Expertise (UE) component. The yellow boxes and blue boxes denote Cluster Attention (CA) component and neural text encoders with attention. The highlighted words in red denoted the text segments that are being attended by the encoder. The ×, $\sum $, and *σ* symbols denote the multiplication, summation, and sigmoid, respectively

**Self-supervised Drug Side Effect Discovery.** We propose a self-supervised learning objective. Instead of relying on the identical and independently distributed assumption of fully supervised learning, we construct the dataset from threads that can discuss a set of common drugs. We look up the side effects of these mentioned drugs via a drug–side effect medical database obtained from Mayo Clinic portal. Our self-supervised task explores discussion-based side effect discovery which alleviates the need for finer-grain annotation compared against existing approach of statement-based side effect discovery. We also propose our neural architecture, NEAT, that jointly models user credibility, expertise and text content with attention while optimizing for the self-supervised objective. The network has three major components: 1) user expertise representation with rich multi-dimensional vectors; 2) cluster-sensitive attention being capable of focusing on relevant phases for post content encoding improvement; and 3) credibility weighting mechanism which effectively learns to assign credibility score to each user, based on their content. We discuss its implementation in the following sections. Figure 1 shows the detailed network architecture of our model.

**Fig. 2:**
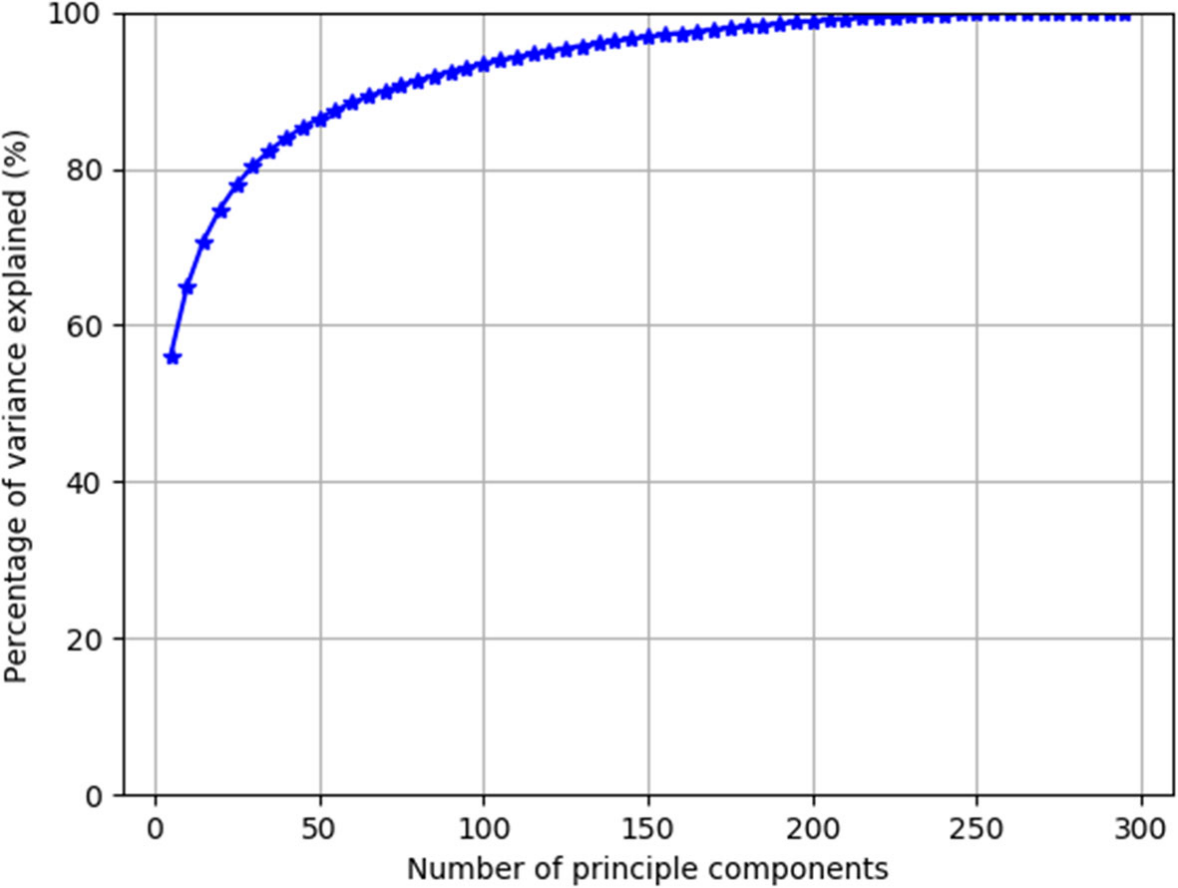
Principal component analysis on user experience vectors. The horizontal axis denotes the number of principal components chosen for PCA, while the vertical axis denotes their percentage of variance explained. We notice that the percentage of variance explained does not increase significantly after 100 principal components

**User Expertise Representation (UE).** We embed each user *u*∈*U* as a vector ***v***_*u*_ so that the vector captures user *u*’s experience with certain side effects. As each user *u* participates in the threads *T*_*u*_, entailing a list of experienced side effects, we derive user side effect experience vector $\boldsymbol {v^{\ast }}_{u} \in \mathbb {R}^{|S|}$ where *S* is the set of all possible side effects and $v^{\ast }_{u_{i}}=n_{u_{i}}$ where user *u* has discussed *i*^*th*^ side effect in $n_{u_{i}}$ threads. We obtain a user drug experience matrix $\boldsymbol {M}^{\ast } \in \mathbb {R}^{|U|\times |S|}$ where *j*^*th*^ row of ***M***^∗^ denotes user side effect experience vector of *j*^*th*^ user. To avoid learning from sparse multi-hot encoded representations and to improve the model’s scalability with the number of side effects, we perform dimensionality reduction, specifically principal component analysis (PCA) [37], to our experience matrix ***M***^∗^ obtained from training set. Figure 2 shows percentage of variance explained versus number of included principal components. Since our PCA plots do not show significant improved percentage of variance explained beyond 100 components, we use *g*=100 components, reducing our original $\boldsymbol {M}^{\ast }\in \mathbb {R}^{|U|\times |S|}$ to user expertise matrix $\boldsymbol {M} \in \mathbb {R}^{|U| \times g}$.

**Fig. 3:**
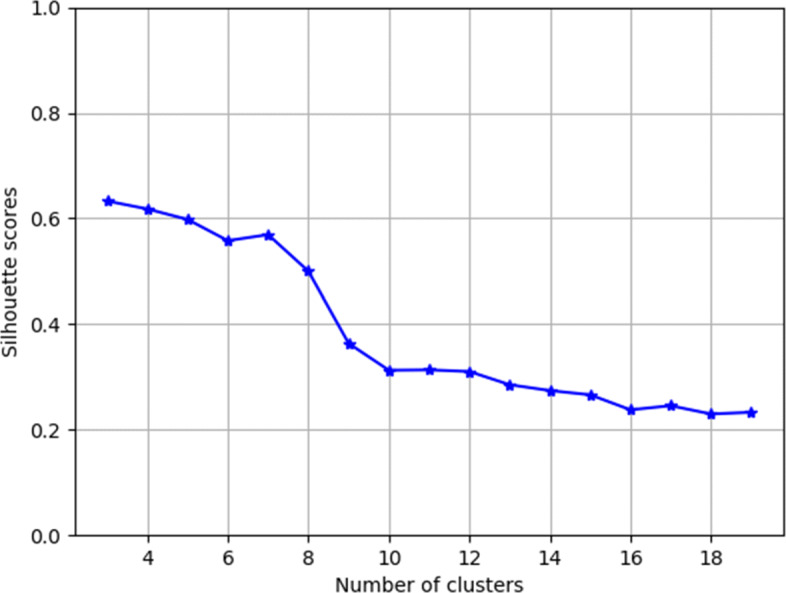
Silhouette scores for User Clustering. The horizontal axis denotes the number of clusters chosen for *K*-means clustering, while the vertical axis denotes their the silhouette scores. We notice that the silhouette scores drop sharply after 7 clusters.

**User Cluster Attention (CA).** We make an assumption via observations that users in online health communities can be effectively grouped into clusters based on their previous side effect experience. The advantages of clustering users is twofold: First, since users in the same clusters share certain parameters, they are jointly modeled and more active forum members leverage less active ones. Second, clustering efficiently reduces the number of parameters to learn and improves optimization and generalization. We apply *K*-means – a distance-based unsupervised clustering algorithm [38] – to binary-valued user experience vectors $\boldsymbol {v^{*}_{u}}$ after normalization. By using cosine similarity, the algorithm effectively groups users with a high number of co-occurred side effects in the same cluster. To determine the number of clusters *c*, we plot the silhouette scores against the number of clusters and observe the sharp drop after *c*=7 (Fig. 3). The average silhouette score is 0.57 for our choice of *c*=7, indicating that users are moderately matched to their own groups and separated from other groups. The top 5 most common side effects in each clusters are shown in Table 3.

**Table 3:** Most common experienced side effects for each user cluster *c*_*i*_ (*i*=1 to 7)

Cluster	Most common experienced side effects
*c*_1_	vision blurred, yellow skin, vision double, yellow eye, nose stuffy
*c*_2_	headache, itch, stomach pain, weak, nausea
*c*_3_	itch, irritate, headache, pain abdominal, stomach cramp
*c*_4_	bad taste, nausea, tiredness, irritate, mouth ulcer
*c*_5_	skin red, itch, rash skin, skin peeling, burning skin
*c*_6_	sneezing, nose runny, nose stuffy, decrease sexual desire, pain breast
*c*_7_	nausea, stomach pain, vomit, diarrhea, pain abdominal

The left column lists the names of 7 clusters, and the right column describes the most common experienced side effects of users in each cluster

In the larger domain of natural language processing, attention has become an integral part for modeling text sequences [39, 40]. By learning to focus on essential text segments, attention allows text encoders to capture long term semantic dependencies with regard to auxiliary contextual information [41, 42]. In our related task of ADR mentions extraction, attention has been adopted recently in neural sequence labelling models [21, 43], resulting in promising improvement. Inspired by the concept, we enhance text encoding with user expertise attention. Even though the attention is adjusted to the non-extractive self-supervised task of thread-level drug side effect discovery, we hypothesize that our model learns to highlight the mentioned accurate side effects, and can be used as a self-supervised baseline for side effect extraction. Based on the previously obtained clustering results, we assign a learnable cluster attention vector for each user group and incorporate their expertise into the text encoding process.

**Fig. 4:**
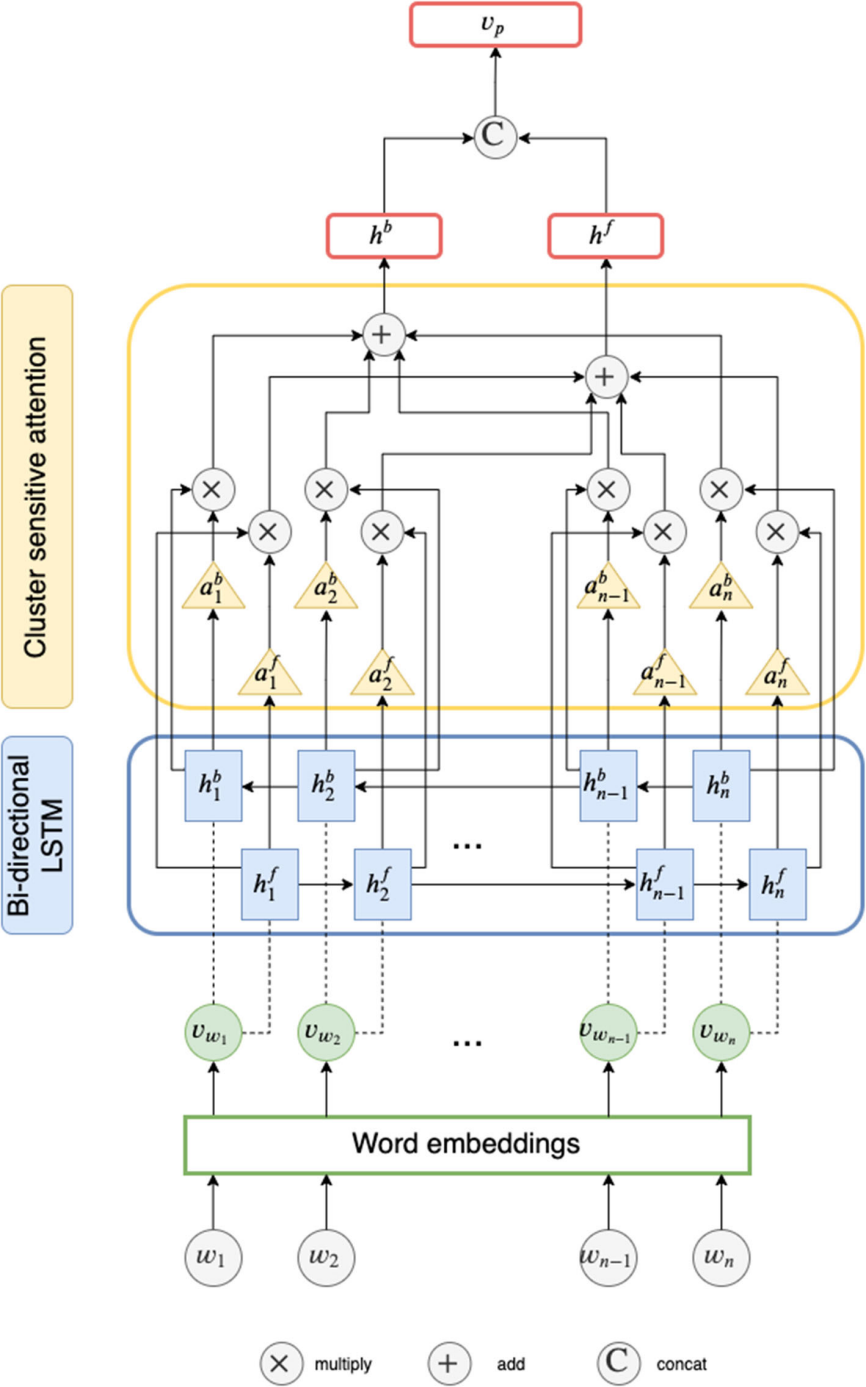
LSTM-based encoder with cluster attention. The × and + cells denote the attention-weighted summation described in Eq. (2). The C cell denotes the concatenation of the forward, ***h***^***f***^, and backward, ***h***^***b***^, hidden states

**Fig. 5:**
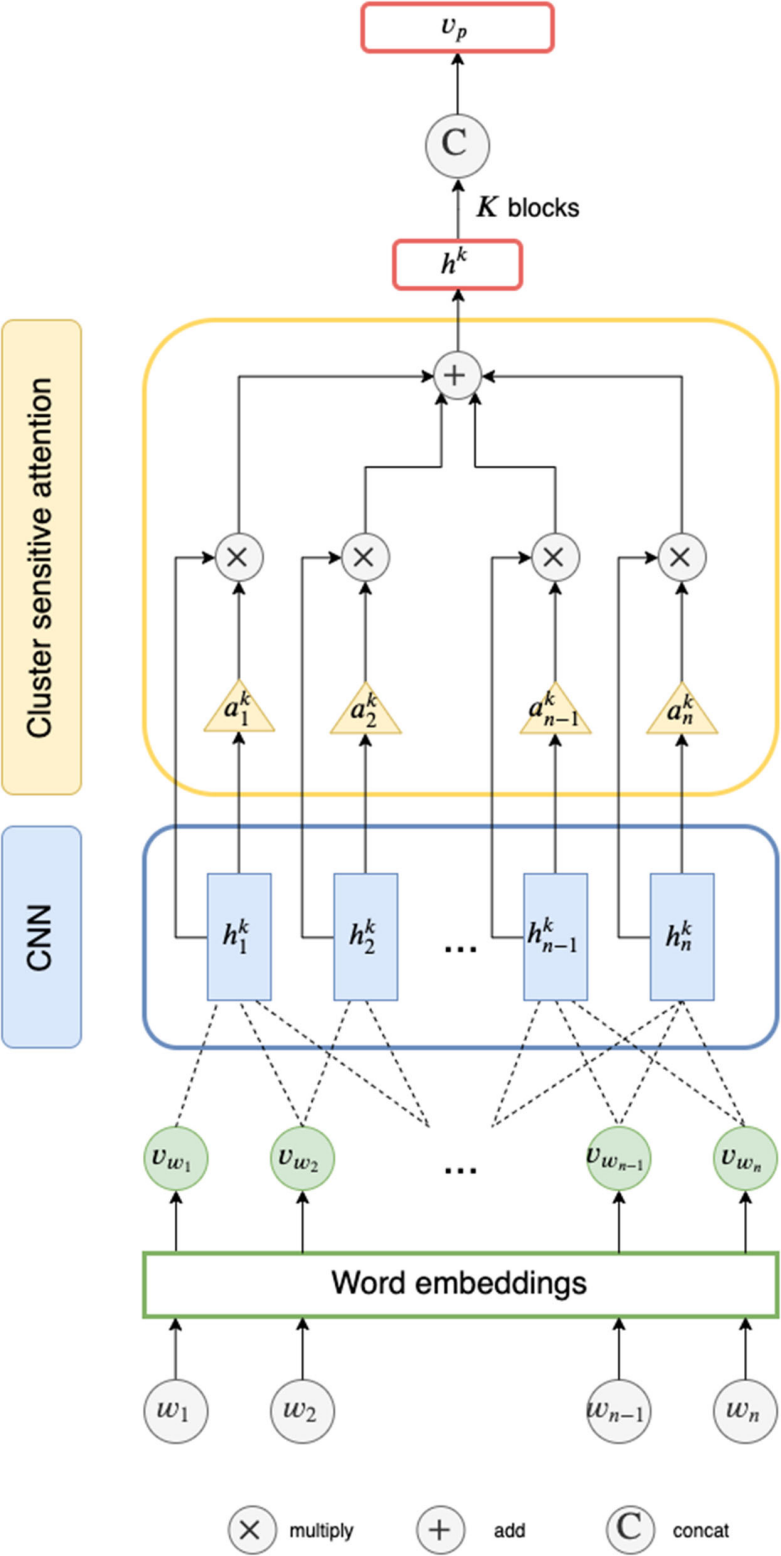
CNN-based Encoder with Cluster Attention. The × and + cells denote the attention-weighted summation described in Eq. 2. The C cell denotes the concatenation of the final hidden states of *K* convolution blocks

**Post Content Encoding.** NEAT takes the content of a thread *t* as input, which is a list of post–user pairs *Q*_*t*_. Post *p*_*i*_ of pair (*p*_*i*_,*u*_*i*_)∈*Q*_*t*_ consists of a sequence of words $x_{p_{i}}$ = {*w*_1_,…,*w*_*n*_} with length *n*. We seek to represent a post *p*_*i*_ as a vector ***v***_*p*_ that effectively captures its semantics through an encoding function $f(x_{p_{i}})$ modeled by a neural text encoding module (the blue boxes in Fig. 1). We embed each word into a low dimensional vector and transform the post into a sequence of word vectors $\{\boldsymbol {v}_{w_{1}}, \boldsymbol {v}_{w_{2}},\ldots, \boldsymbol {v}_{w_{n}}\}$. Each word vector is initialized using pre-trained GloVe [24] embeddings, and each out-of-vocabulary word vector is initialized randomly. We make use of modularity – a major advantage of neural architectures – and design the post content encoder as a standalone component that can be easily updated with any state-of-the-art text encoder. In this work, we provide two neural text encoders: long-short term memory (LSTM, see Fig. 4) [36] and convolutional neural networks (CNN, see Fig. 5) [18], both of which incorporates attention mechanism.

A bi-directional LSTM encodes the word vector sequence and outputs two sequences of hidden states: a forward sequence, $H^{f}={\boldsymbol {h}^{f}_{1}, \boldsymbol {h}^{f}_{2},\ldots, \boldsymbol {h}^{f}_{n}}$ that starts from the beginning of the text; and a backward sequence, $H^{b}={\boldsymbol {h}^{b}_{1}, \boldsymbol {h}^{b}_{2},\ldots, \boldsymbol {h}^{b}_{n}}$ that starts from the end of the text. For many sequence encoding tasks, knowing both past (left) and future (right) contexts has proven to be effective [44]. The states $\boldsymbol {h}^{f}_{i},\boldsymbol {h}^{b}_{j}\in \mathbb {R}^{e}$ of the forward and backward sequences are computed as follows:

$$\begin{array}{@{}rcl@{}} \boldsymbol{h}^{f}_{i}=LSTM(\boldsymbol{h}^{f}_{i-1}, \boldsymbol{v_{w_{i}}}), \hspace{1mm}\boldsymbol{h}^{b}_{j}=LSTM(\boldsymbol{h}^{b}_{j+1}, \boldsymbol{v_{w_{j}}}),  \end{array} $$

where *e* is the number of encoder units, and $\boldsymbol {h}^{f}_{i}, \boldsymbol {h}^{b}_{j}$ are the *i*th and *j*th hidden state vector of the forward (*f*) and backward (*b*) sequence. We derive the cluster attention vector as $\boldsymbol {v}_{a_{i}}\in \mathbb {R}^{e}$ for each user *c*_*i*_, from which the weights of each hidden state $\boldsymbol {h}^{f}_{j}$ and $\boldsymbol {h}^{b}_{j}$ based on their similarity with the attention vector are:

1 $$\begin{array}{@{}rcl@{}} w_{a_{j}}&=&\frac{exp(\boldsymbol{v}_{a_{i}} \boldsymbol{h}_{j})}{\sum^{n}_{l=1}exp(\boldsymbol{v}_{a_{i}} \boldsymbol{h}_{l})}. \end{array} $$

The intuition behind Eq. (1), inspired by Luong et al. [39], is that hidden states which are similar to the attention vector $\boldsymbol {v}_{a_{i}}$ should be paid more attention to; hence are weighted higher during document encoding. $\boldsymbol {v}_{a_{i}}$ is adjusted during training to capture hidden states that are significant in forming the final post representation. $w_{a_{j}}$ is then used to compute forward and backward weighted feature vectors:

2 $$\begin{array}{@{}rcl@{}} \boldsymbol{h}^{f}=\sum^{n}_{j} w_{a_{j}} \boldsymbol{h}^{f}_{j}, \quad\boldsymbol{h}^{b}=\sum^{n}_{j} w_{a_{j}} \boldsymbol{h}^{b}_{j}. \end{array} $$

We concatenate the forward and backward vectors to obtain a single vector, following previous bi-directional LSTM practice [45].

Our choice of CNN-based encoder is based on prior work [18, 46]. A convolution block *k* consists of two sub-components: a convolution layer and a cluster attention layer. In the convolution layer, a kernel of window *s* (0<*s*<*n*) of weight ***W*** is used to generate the hidden representation $\boldsymbol {h^{k}_{j}}$ for the word embeddings $\{\boldsymbol {v_{w_{i-s+1}}},\cdots,\boldsymbol {v_{w_{i}}}\}$ as:

3 $$ \boldsymbol{h^{k}_{j}} = CONV(\boldsymbol{W}, \{\boldsymbol{v_{w_{i-s+1}}},\cdots,\boldsymbol{v_{w_{i}}}\})  $$

where *CONV*(·) is the convolution operation described in [18]. In the cluster attention layer, we first derive the attention weight $w_{a_{j}}$ for each hidden representation $\boldsymbol {h^{k}_{j}}$ similarly to the LSTM-based encoder. Attention weighted pooling is used to obtain the convolution block output as follows:

4 $$ \boldsymbol{h}^{k}=\sum^{n}_{j} w_{a_{j}} \boldsymbol{h}^{k}_{j}  $$

Since we use multiple convolution blocks of different kernel sizes, the final post representation is the concatenation of *K* block outputs ***h***^*k*^.

**Thread Content Encoding with Credibility Weights (CW).** For every post–user pair (*p*_*i*_,*u*_*i*_) at thread *t*, we first compute feature vector $\boldsymbol {v}_{p_{i}}$ for post *p*_*i*_. NEAT then concatenates this post–user representation with user *u*_*i*_’s expertise vector $\boldsymbol {v}_{u_{i}}$ to form post–user complex vector $\boldsymbol {v}^{p}_{u_{i}}$. This post–user complex is weighted by a user credibility $e^{w_{u_{i}}}$, where $w_{u_{i}}$ initially set to 0 per user and updated while training for the self-supervised side effect discovery objective. We implement credibility learning according to the general intuition from the truth discovery literature: users who give quality posts, on which the model can solely base to make correct predictions, are given a higher credibility. We also exploit this credibility score to encode the thread representation by placing emphasis on the content of credible users. A representation of a thread that meets the above description is the weighted sum of each post–user complex vector:

5 $$ \boldsymbol{v}_{t} = \sum^{n}_{i=1} \boldsymbol{v}^{p\ast}_{u_{i}} = \sum^{n}_{i=1} e^{w_{u_{i}}} \boldsymbol{v}^{p}_{u_{i}}  $$

**Multi-label Prediction:** NEAT feeds the thread content representation *v*_*t*_ through a fully connected layer whose outputs can be computed as follows:

6 $$ \boldsymbol{s}_{t}=\boldsymbol{W} tanh(\boldsymbol{v}_{t})+\boldsymbol{b},  $$

where ***W*** and ***b*** are weights and biases of the layer. The output vector $\boldsymbol {s}_{t} \in \mathbb {R}^{|S|}$ is finally passed through a sigmoid activation function *σ*(·), and trained using cross-entropy loss *L* defined as follows:

7 $$ {}\begin{aligned} L=\frac{1}{|T|}\sum\limits_{t=1}^{|T|}\{\boldsymbol{y}_{t} \cdot log(\sigma(\boldsymbol{s}_{t}))&+(1-\boldsymbol{y}_{t})\cdot log(1-\sigma(\boldsymbol{s}_{t}))\}& \\ &+\lambda_{1}\sqrt{\sum_{u}\boldsymbol{v}_{u}^{2}}+\lambda_{2}\sum_{i}{{|\boldsymbol{w}_{u_{i}}|}}& \end{aligned}  $$

We adopt regularization that penalizes the training loss with the user experience matrix’s *L*2 norm by a factor of *λ*_1_ and the user credibility vector ***w***_*u*_’s *L*1 norm by a factor of *λ*_2_. The loss function is differentiable, thus trainable with the Adam optimizer [47]. During our gradient-based learning, user *u*_*i*_’s credibility score $w_{u_{i}}$ is updated by calculating $\frac {\partial L}{\partial w_{u_{i}}}$ by back-propagation (see Appendix 1).

## Results

We conduct experiments to validate the effectiveness of our proposed model. We design an ablation study to highlight the effectiveness of each component of NEAT in our self-supervised side effect prediction. In addition, we expand our previous work [16]. More specifically,

1. We verify the representativeness of the learned credibility scores via correlation analysis and ranking metrics using number of “thanks” received by other forum members as the trustworthiness proxy.
2. We compare the model’s performance in unseen drug side effect discovery against non-neural baselines.
3. We examine the applicability of cluster attention in side effect mention extraction from user posts both at the macroscopic and microscopic levels.

**Dataset and Experiment Settings.** We conduct our experiments on the same dataset as [13] including 15,000 users and 2.8 million posts extracted from 620,510 HealthBoards ^[1]^ threads. The ground truths for self-supervised learning are defined as side effects of mentioned drugs in the discussion. As annotating such amount of posts is expensive, drug side effects are extracted from Mayo Clinic’s Drugs and Supplements portal^3^ and are used as surrogates for potential drug reactions. From the original dataset, we only extract threads that are annotated with drugs and their side effects, along with the lists of contained posts and corresponding users. Table 4 shows some statistics of our dataset.

**Table 4:** Some statistics on our dataset

# Users	14,966
# Threads	78,213
Avg. words per post	67.45
Avg. posts per thread	3.97
Avg. participated threads per user	54.7
# Side effects (SE)	315
Avg. SEs per thread	74.25
# Drugs	1869
Avg. experienced side effects per user	128.12

The left column contains the statistics’ descriptions while the right column contains the statistics’ values

For CNN encoder, we adopt the work by Kim (2014) [18] and use three kernels of sizes 3, 4, 5 with output channel size = 100. For Bi-LSTM we use a single layer with a hidden state size = 32.

We used Natural Language Toolkit 4 for tokenization and stop-word elimination before representation modeling. We perform 10-fold cross-validation (with 8:1:1 folds for training, validation, and testing, respectively). We perform PCA and *K*-means clustering on training set, using scikit-learn’s built-in modules [48], 100 principal components (*g*=100). All models are trained using PyTorch^5^ library. We have released our codes at 6.

**Ablation Study.** We include each component in Section 1 to the architecture at a time and verify the incremental enhancement. We implement both CNN and LSTM-based text encoders to confirm the consistent improvement across different neural encoders. The ablated baselines and the full models are as follows:

- **Vanilla**: We implement a neural text encoder baseline without any proposed component.
- **Weighted Post Encoder (WPE)**: We construct thread representation by summing each of its post–user complex vector weighted by user credibility.
- **Weighted Post Encoder with User Expertise (WPEU)**: We concatenate user expertise and post vector to create post–user complex vector.
- **NEAT**: We incorporate all three components – UE, CW and CA – as described.

Table 5 shows the precision, recall (sensitivity), and *F*_1_ (the harmonic mean of precision and sensitivity) obtained by our method and the four baselines. We also report the performance of baselines implementing UE and CA individually in Table 11.

**Table 5:** Performance of CNN-based models and LSTM-based models in Ablation Study

**Systems**	Components			Evaluation Metrics		
	CW	UE	CA	Pre.	Rec.	*F*_1_
LSTM-Vanilla				0.6173	0.407	0.4335
LSTM-WPE	✓			**0.6376**	0.4344	0.4503
LSTM-WPEU	✓	✓		0.6064	0.5001	0.4896
LSTM-NEAT	✓	✓	✓	0.6197	**0.5134**	**0.5064**
CNN-Vanilla				0.7214	0.5503	0.5637
CNN-WPE	✓			**0.7423**	0.5799	0.5804
CNN-WPEU	✓	✓		0.6923	0.6350	0.5910
CNN-NEAT	✓	✓	✓	0.7066	**0.6431**	**0.6139**

In the Components column, CW, UE, CA denote Credibility Weights, User Expertise and Cluster Attention module components, respectively. In the Evaluation Metrics column, Pre., Rec. and *F*_1_ denote Precision, Recall, and *F*_1_ score

**User Credibility Analysis.** We discuss how descriptive the credible users assigned by the model are to our common notion of trustworthy users in online communities. We employ the number of “thanks” received by other community members as the proxy for a user’s credibility, at both global, forum-wise scope and local, thread-wise scopes. Specifically, at forum-wise scope, we measure Spearman’s rank correlation coefficient to examine how our output user scores approximate the ordering of user trustworthiness. Thread-wise, we examine how accurate our output user scores are in ranking trustworthy respondents within a single discussion by measuring both Spearman’s coefficient and nDCG@2 with regard to the ordering provided by our credibility proxy. We find the forum-wise ranking metrics meaningful as answer ranking, based on user credibility in our case, is a well-formulated task in CQA [49–51]. The measurement results are presented in Table 6.

**Table 6:** Analysis of NEAT’s Credibility versus baselines in approximating credibility proxy

**Methods**	Thread nDCG@2	Thread Spearman	Forum Spearman
Random	0.7968	-0.0271	0.0
Post frequency	0.8812	0.4223	0.1924
Question frequency	0.8341	0.1773	0.0279
NEAT’s Credibility	**0.8856**	**0.4403**	**0.3055**

The Thread nDCG@2, Thread Spearman, and Forum Spearman columns respectively denote the values of Normalized Discounted Cumulative Gain at 2 at thread level, Spearman’s rank correlation coefficient at thread level and forum level of each method when using rankings by number of thanks as ground truths

**Drug Side Effect Discovery.** We test the performance of NEAT in the task of Drug Side Effect Discovery. Specifically, the model has to predict the side effects of one of five unseen drugs based on their discussions as a whole. Such task is necessary to verify that our self-supervised objective of predicting for side effects of discussed drugs generalizes well to drugs that have not been discussed in training data. We highlight the performance of an end-to-end neural architecture against Random Forest (RF) – a competitive, non-neural baseline trained on bag-of-word text representations of thread content. Additionally, we examine a baseline, uNEAT, where the user identities are randomized in order to verify that NEAT effectively considers both user credibility and expertise. The results of drug side effect discovery on Ibuprofen, Levothyroxine, Metoformin (Table 7), and Omeprazole, Alprazolam (Table 8) are reported.

**Table 7:** Performance of NEAT versus baselines in Side Effect Discovery of Ibuprofen, Levothyroxine, and Metoformin

**Methods**	Ibuprofen			Levothyroxine			Metoformin		
	Pre.	Rec.	*F*_1_	Pre.	Rec.	*F*_1_	Pre.	Rec.	*F*_1_
RF	0.583	0.414	0.474	0.319	0.401	0.347	0.48	0.647	0.491
uNEAT	0.859	0.371	0.487	0.505	0.349	0.404	0.798	0.361	0.497
NEAT	0.845	0.427	**0.536**	0.549	0.385	**0.443**	0.814	0.365	**0.504**

In the Methods column, RF denotes Random Forest baseline from Bag-of-word, and uNEAT denotes User permutation baseline from NEAT. Pre., Rec. and *F*_1_ denote Precision, Recall, and *F*_1_ score, respectively.

**Table 8:** Performance of NEAT versus baselines in Side Effect Discovery of Omeprazole and Alprazolam

**Methods**	Omeprazole			Alprazolam		
	Pre.	Rec.	*F*_1_	Pre.	Rec.	*F*_1_
RF	0.229	0.458	0.271	0.639	0.432	0.511
uNEAT	0.534	0.393	0.394	0.981	0.551	0.663
NEAT	0.522	0.421	**0.41**	0.977	0.596	**0.704**

In the Methods column, RF denotes Random Forest baseline from Bag-of-word, and uNEAT denotes User permutation baseline from NEAT. Pre., Rec. and *F*_1_ denote Precision, Recall, and *F*_1_ score, respectively

**Side Effect Extraction with Cluster Attention.** As discussed in Section 1, our employed attention mechanism not only offers a better textual encoding capacity but also locates the informative segments of user-generated content. This concept is well-aligned with ADR mention extraction, which is mainly modeled as a sequence labeling problem. We conduct experiments to examine the effectiveness of CNN-NEAT’s Attention in locating the text segments containing the correct side effects. We benchmark our results against a lexicon-based tagging using UMLS thesaurus for medical terms [52] and a state-of-the-art neural side effect extractor [21] which was supervisedly trained to identify side effect mentions in social media contents. In this task, correctly predicting the positive side effects is of the utmost importance, hence, we benchmark the text segments extracted from CNN-NEAT’s Attention against two mentioned baselines on precision metric. The experiment results on Ibuprofen, Levothyroxine, Metoformin, Omeprazole and Alprazolam are reported in Table 9.

**Table 9:** Performance of CNN-NEAT’s Attention versus baselines in Side Effect Extraction in term of Precision

**Methods**	Ibuprofen	Levothyroxine	Metoformin	Omeprazole	Alprazolam
UMLS Tagging	0.6801	0.6145	0.8378	**0.5218**	0.614
Neural Extractor [21]	0.6741	0.6259	0.8092	0.4665	0.6161
CNN-NEAT’s Attention	**0.7073**	**0.7119**	**0.8557**	0.504	**0.688**

The Methods column includes the two baselines, UMLS Tagging and Neural Extractor, and the extracted attention of our proposed NEAT – CNN-NEAT’s Attention. We present the five evaluated drugs, Ibuprofen, Levothyroxine, Metoformin, Omeprazole, Alprazolam and the Precision of extracting their side effects for all three methods.

## Discussion

**Ablation Study.** Firstly, all of the three models that apply credibility weighting (CW) – WPE, WPEU, and NEAT — outperform both LSTM-Vanilla and CNN-Vanilla baselines. Specifically, in LSTM-Vanilla, solely weighting each post by its author credibility improves the performance of the naive post encoder by 2.03%, 2.74% and 1.68% on precision, recall, and *F*_1_, respectively. We observe a similar margin of improvement from CNN-Vanilla. These results demonstrate the effectiveness of accounting for author credibility when encoding thread content, improving side effect prediction.

Improvements by incorporating user experience (UE) are also testified in both neural encoders. In LSTM-based models, adding UE (LSTM-WPEU vs. LSTM-WPE) improves recall by 6.57% and 3.93% in *F*_1_. Again, the CNN-based counterpart, CNN-WPEU, shows similar performance trends. On a macro scale, these statistics indicate that our model successfully learns to include more side effects in its prediction, where many are relevant to the ground truth. This is consistent with our hypothesis that considering author experience of each post is effective in predicting out-of-context side effects.

Applying cluster-sensitive attention (CA) in combining both the LSTM’s and CNN’s hidden states also improves the performance. In LSTM-based systems, we observe that adding CA (LSTM-NEAT vs. LSTM-WPEU) uniformly improves all retrieval metrics; our CNN-based counterpart, CNN-NEAT, also demonstrates similar performance improvements. Although CNN-NEAT and its ablated baselines obtain higher performance than the LSTM counterparts, when measuring relative improvement, the gains are comparable. This confirms the consistent improvement of our proposed components across different neural encoders. According to the macroscopic analysis of results in Table 5, we generally conclude that all of the three components in our proposed architecture – namely, CW, UE, and CA – yield a positive impact on the overall model performance. We observe consistent improvements in *F*_1_ after adding each component, and this lends support our stated hypotheses. The significance of these findings were verified by one-tail *t*-test of *p*<0.05.

**User Credibility Analysis.** Results at both the thread and forum level show that user scores assigned by NEAT reasonably approximate our credibility proxy, i.e. the number of “thanks” given by other forum users. Regarding ranking users by their helpfulness within a thread, NEAT’s credibility improves heuristics such as post or question frequency marginally by 0.0044 in term of nDCG@2 and moderately by 0.0180 in term of Spearman’s coefficient. Regarding ranking users by their helpfulness in the whole forum, we report a more significant improvement of 0.1131 in term of Spearman’s coefficient from the closest performing baseline of post frequency. These results verify the representativeness of the credibility scores obtained in the self-supervised manner by our system.

**Drug Side Effect Discovery.** Both neural methods, namely uNEAT and NEAT, outperform non-neural approach based on bag-of-word vectors on all metrics and across all drugs. Specifically, NEAT improves RF by 9.94% in *F*_1_ on average across five different drugs. This confirms the modeling capability of our neural network and justifies its application to our task. We also observe a decrease in performance while optimizing NEAT being user-unaware. This shortcoming is prevalent in all side effect discovery settings, ranging from a 0.7% F1 score decrease for Metformin to 4.9% decrease for Ibuprofen. Overall, being aware of user experience and expertise improves drug side effect discovery by 3.04% in *F*_1_ on average across five different drugs. This gives substantial indicative evidence congruent with our hypothesis that considering the credibility and experience of users improves drug side effect discovery performance in online health communities.

**Side Effect Extraction with Cluster Attention.** We notice improvement of CNN-NEAT’s Attention from both baselines across most drugs with the exception of Omeprazole. At a macro level, positive precision scores confirm CNN-NEAT’s emphasis on critical text segments, i.e. those containing correct drug side effects. The significant improvement in most cases also suggests the selectiveness of CNN-NEAT’s Attention. Unlike the two proposed baselines which extract any side effect mentions, CNN-NEAT’s Attention selectively emphasizes correct ADRs. We also examine this hypothesis at the micro level in Table 10. UMLS tagging identifies any phrase having a medical nuance, i.e. *pain* and *back*, and potentially forms side effects that are not actually mentioned, i.e. *back pain*, whereas both neural methods, Neural Extractor and NEAT, that model textual semantics are able to dismiss such trivial mentions. *Discomfort*, although correct, is questionably an intentionally reported side effects and is also dismissed by both Neural Extractor and CNN-NEAT. Post-processing rules can arguably alleviate the shortcomings of UMLS’s matching strategy due to missing context awareness. However, such rules are not efficient to engineer. On the other hand, unsupervised context encoding for keyword-based extraction is challenging, and the task is left open for future works. We attempt to explain CNN-NEAT’s decision to dismiss *restlessness* based on its contextual awareness. The attention was derived from each cluster’s experienced side effects, in which there is the *weak* side effects being semantically contradicting to *restlessness*. Although having not fully covered all side effects, all mentions extracted by CNN-NEAT are accurate, giving it the highest precision amongst the three considered models. Specifically, CNN-NEAT’s Attention improves over neural ADR extraction model [21] by 5.502% and UMLS tagging by 3.974% on average in terms of precision across five different drugs. Despite being derived from a self-supervised objective, CNN-NEAT’s Attention offers helpful indications for attention-based models and a strong baseline for ADR extraction.

**Table 10:** A test example highlighting the extracted side effects obtained by CNN-NEAT’s Attention versus baselines

	

In the Post content column, the correct and incorrect side effects are highlighted in blue and red, respectively. The extracted side effects of UMLS Tagging, Neural Extractor and CNN-NEAT’s Attention are followed by <*U*>, <*X*>, and <*N*>, respectively. The Cluster’s side effects column shows the list of common side effects in user 8420’s cluster

**Table 11:** Performance for individual integration of UE and CA in Ablation Study

**Systems**	Components			Evaluation Metrics		
	CW	UE	CA	Pre.	Rec.	*F*_1_
LSTM-UE		✓		0.6513	0.4204	0.4531
LSTM-CA			✓	0.6416	0.4293	0.4611
CNN-UE		✓		0.6738	0.6185	0.5743
CNN-CA			✓	0.7441	0.5616	0.5883

In the Components column, CW, UE, CA denote Credibility Weights, User Expertise and Cluster Attention module components, respectively. In the Evaluation Metrics column, Pre., Rec. and *F*_1_ denote Precision, Recall, and *F*_1_ score

**Limitations.** We call attention to limitations from our design choice of defining a user’s credibility by Eq. (5), as well as our choice of credibility proxy as defined by the number of “thanks”. A user’s credibility can be damaged if their posts do not directly help with predicting the correct side effects. This assumption is questionable when users are asking for some information instead of giving answers without any intent to give misleading information. In contrast, we also observe the cases where users receive thanks for giving helpful information such as suggesting nutritious diet or healthy lifestyle without mentioning any relevant side effects. We recognize the limitation of our model where users without malicious intent are possibly assigned a low credibility score. This case of “failure” can explain why some users are assigned low to moderate credibility despite their high number of “thanks”. However, our definition makes sure that the credibility learning mechanism does not express the opposite adverse behavior of assigning high credibility to untrustworthy users.

Annotating a post’s side effects solely from looking up the mentioned drugs in the Mayo Clinic database presents another limitation. To generalize the usability and ensure the effectiveness of the learning framework to a broader community such as medical informatics, we suggest to have these annotations cross-checked with healthcare professionals or pharmacovigilance experts, in order to ensure the correlation between Mayo Clinic’s annotations and the post’s actually described side effects.

Overall, our analysis suggests that user credibility scores, although learned in a self-supervised manner, can capture the expected notion of credibility and are descriptive of trustworthiness. Every component of our architecture is also shown to be vital in achieving the highest performance.

## Conclusion

We have addressed the importance of user experience and credibility in modeling thread contents of online communities, specifically through the task of drug side effect discovery. Our proposed neural architecture, NEAT, suggests a subset of side effects relevant to the mentioned treatment in the given discussion, while taking into account the each post content and its author side effect experience via attention mechanism to represent forum discussions better. Mainstream models for drug discovery in online communities have not captured thread content and user experience holistically in an end-to-end optimizable system.

We modeled users’ expertise by examining their experience with different side effects, and then grouped the users with similar experience into clusters that share a common attention representation. We also proposed an self-supervised method which assigns credibility scores to users based on the correctness of their contents and overall improves thread representations. Correlation analysis testifies the representativeness of learned credibility scores of trustworthiness approximated by number of “thanks” received by other online community members. In addition, our integrated attention mechanism not only enhances textual encoding but also highlights essential text segments and benefits ADR extraction approaches.

We believe that our model is applicable to other domains. We plan to generalize its application to mainstream CQA or expertise-based thread recommendation for health forum members.

## Appendix

### A User credibility weighting and the general principle of truth discovery

In order to demonstrate the correlation between learned user credibility scores and the general notion of trustworthiness, we derive how user credibility scores are updated after each turn of back-propagation via stochastic gradient descent. The overall loss function in Eq. (7) can be rewritten as logistic loss without regularization on a single training example and a single label *s* as follows:

8 $$ L = log(1+exp(-y_{s}(\boldsymbol{w}^{\top}_{s} tanh(\boldsymbol{v_{t}})+b_{s}))),  $$

where *y*_*s*_ is the binary truth for label *s*, *b*_*s*_ is a classification bias, and $\boldsymbol {w_{s}}\in \mathbb {R}^{g\times 1}$ is a row of ***W*** in Eq. (6). ***w***_*s*_ is the classification weight vector of a single label *s*.

In back-propagation, we update the score $w_{u_{i}}$ of user *u*_*i*_ based on the gradient calculated by taking the derivative of the loss *L* with regard to $w_{u_{i}}$:

9 $$ \frac{\partial L}{\partial w_{u_{i}}} = \frac{(1-tanh(\boldsymbol{v_{t}})^{2}){y_{s}}\boldsymbol{w}^{\top}_{s} \boldsymbol{v}^{p}_{u_{i}}e^{w_{u_{i}}}}{1+exp(y_{s}(\boldsymbol{w}^{\top}_{s} tanh(\boldsymbol{v_{t}})+b_{s}))}=\nabla_{w_{u_{i}}}L.  $$

The user score $w_{u_{i}}$ is updated as follows:

10 $$ w^{t+1}_{u_{i}} = w^{t}_{u_{i}} - \eta\nabla_{w^{t}_{u_{i}}}L,  $$

where *η* is the learning rate.

When the prediction is correct, *y*_*s*_ and $(\boldsymbol {w}^{\top }_{s} tanh(\boldsymbol {v_{t}})+b_{s})$ share the same sign and $y_{s}(\boldsymbol {w}^{\top }_{s} tanh(\boldsymbol {v_{t}})+b_{s}))$ is highly positive, making the denominator highly positive and the overall gradient small. The user score $w_{u_{i}}$ is minimally updated.

When the prediction is incorrect, *y*_*s*_ and $(\boldsymbol {w}^{\top }_{s} tanh(\boldsymbol {v_{t}})+b_{s})$ have different signs and the denominator approaches 1. In the nominator, $\boldsymbol {w}^{\top }_{s} \boldsymbol {v}^{p}_{\boldsymbol {u_{i}}}$ is the prediction if we solely consider the post vector $\boldsymbol {v}^{p}_{u_{i}}$ of user *u*_*i*_.

- If this prediction is correct, which fits our definition of credible user, ${y_{s}}\boldsymbol {w}^{\top }_{s} \boldsymbol {v}^{p}_{u_{i}}$ is positive, making the overall gradient positive. Then, the user score $w_{u_{i}}$ is updated in the positive direction and the credibility score $e^{w_{u_{i}}}$ used to weight user *u*_*i*_’s content increases.
- On the other hand, when the prediction from solely considering the post vector $\boldsymbol {v}^{p}_{u_{i}}$ of user *u*_*i*_ is incorrect, indicating a not credible user, ${y_{s}}\boldsymbol {w}^{\top }_{s} \boldsymbol {v}^{p}_{u_{i}}$ is negative, and the overall gradient is negative. $w_{u_{i}}$ is updated in the negative direction and the credibility score $e^{w_{u_{i}}}$ used to weight user *u*_*i*_’s content decreases.
- The magnitude of the gradient is proportional to $e^{w_{u_{i}}}$. This indicates that users who are currently learned as credible are most affected by back-propagation when the model’s prediction is incorrect.

### Algorithm performance for individual integration of UE and CA.

Table 11 reports the performance of baselines implemented UE, and CA individually.

## Data Availability

The dataset analysed during the current study was first published in [13] and is publicly available at https://www.mpi-inf.mpg.de/departments/databases-and-information-systems/research/impact/peopleondrugs/ The source codes of the implementation of this study is publicly available at https://github.com/nguyenvanhoang7398/NEAT The source codes used to generate the results of Side Effect Extraction in Section 1 is available at https://github.com/nguyenvanhoang7398/NEAT/blob/master/adr_extraction.py.

## Abbreviations

Avg: Average
UE: User Expertise Representation
CA: Cluster-sensitive Attention
CW: Thread Content Encoding with Credibility Weights
CNN: Convolutional Neural Network
LSTM: Long-short Term Memory
*F*_1_ score: The harmonic average of the precision and recall
PCA: Principal Component Analysis
#: Number of
NEAT: The neural architecture proposed in this work
uNEAT: The ablated version of NEAT where the user identities are randomized
ADR: Adverse Drug Reaction
RF: Random Forest
nDCG@2: normalized Discounted Cumulative Gain at 2.

## Footnotes

1 https://www.healthboards.com/

2 https://medhelp.org/

3 https://www.mayoclinic.org/drugs-supplements

4 https://www.nltk.org/index.html

5 https://pytorch.org/

6 https://github.com/nguyenvanhoang7398/NEAT
